# Facial expression and oxytocin as possible markers of positive emotions in horses

**DOI:** 10.1038/s41598-018-32993-z

**Published:** 2018-10-02

**Authors:** Léa Lansade, Raymond Nowak, Anne-Lyse Lainé, Christine Leterrier, Coralie Bonneau, Céline Parias, Aline Bertin

**Affiliations:** PRC, INRA, CNRS, IFCE, University Tours, 37380 Nouzilly, France

## Abstract

Behavioural and physiological markers of discrete positive emotions remain little investigated in animals. To characterise new markers in horses, we used tactile stimulations to induce emotional situation of contrasting valence. In the Gentle grooming group (G, N = 13) horses were gently groomed during 11 sessions on the body areas they appreciated the most. Horses in the Standard grooming group (S, N = 14) were groomed using a fixed procedure, reported to induce avoidance reactions in some horses. At session 11, G horses expressed significantly more contact-seeking behaviours than S horses, which expressed significantly more avoidance behaviours. This result suggests positive emotions in G horses and negative emotions in S horses. Blood cortisol, oxytocin, heart rate and heart rate variability never differed between before and after the grooming session. However, after the 11 sessions, basal oxytocin levels were lower in the G than in the S group. This difference was unexpected, but supports studies showing that a low level of basal oxytocin could be a marker of better well-being. Analyses of facial expressions during grooming revealed significant differences between groups. These expressions appear to be more sensitive than behavioural indicators because they alone enabled differentiating emotions according to the group when horses were re-exposed to neutral grooming one year after the treatment.

## Introduction

The study of animal emotion is a field of research that has developed considerably in recent years. In the context of a ‘dimensional approach’, emotions can be characterised according to their two main dimensions^1^: arousal (bodily activation or excitation; e.g. calm versus excited) and valence (negative or positive; e.g. sad versus happy). Long restricted to negative emotions such as fear, present-day work increasingly focuses on the positive side of emotions^2,3^. One of the challenges of research in species which do not share verbal language like humans is to be able to characterise the type of emotion felt by a subject at a given moment. To this end, different kinds of indicators can be used^3^.

The first category concerns behavioural indicators. Some of them can be a reliable source of information regarding the subjective experience of animals. In particular, reward-seeking and avoidance behaviours can notably inform us about the emotional valence of a stimulus^4^. In addition to body language, facial expressions, which are often automatic and very stereotyped^5^ are also well known to convey emotional information in humans and non-human primates. In humans, characteristic facial expressions have been correlated with specific emotions such as fear, anger, joy and surprise^6^. These expressions have been increasingly studied in animals, in particular in species such as primates, dogs and rodents, which like humans, have a large number of facial muscles (review^7^). They have been widely investigated for negative states such as pain (rodents^8^; sheep^9–11^, horses^12,13^) or fear (horses^14^) through grimace scales or during specific activities such as horses being ridden^15^. Facial expressions have also begun to be investigated for positive emotions. A recent study carried out on rodents identified facial indicators of positive emotions during playful manual tickling^16^. The authors revealed that the ears of the rats became more relaxed and pinker during tickling, compared to a negative situation such as exposure to a novel room and white noise. Other studies, particularly in cattle, sheep and horses analysed changes in certain features of the face such as eye shape^17,18^, wrinkled eyes^19^ or ear postures^20–23^. For instance, Proctor & Carder^17^ found that stroking, when perceived positively reduced the percentage of visible eye white in dairy cows. In horses, Hintze *et al*.^19^ reported that the angle between the line through the eyeball and the highest wrinkle decreased during grooming. Studying facial expressions thus seems to be an interesting lead to characterise emotions, and the horse is an appropriate model for at least two reasons. First, the horse benefits from complex face muscles that allow it to express a wide range of facial movements, including many that are also seen in primates^24^. Second, the horse is a social species that establishes sophisticated and long term social relationships, thus requiring effective communication. Given the fact that it is predominantly a visual animal, it is reasonable to think that it uses postural and facial expression to communicate with its conspecifics^24^. In particular, the literature describes a wide range of body postures and some facial expressions that are expressed in social contexts (e.g. snapping, threatening face, etc.^25^). A better understanding of horses’ facial expressions in connection to their emotions could be very helpful to develop new tools to assess and improve their welfare, a key societal challenge.

The second category of emotional indicators concerns physiological markers. These markers have been relatively well investigated regarding negative emotions. For example, rises in heart rate parameters or in cortisol levels have often been described in response to stress-inducing situations (farm animals^26^, horses^27,28^). However, little research has described the relationship between physiological responses and positive emotions. A few studies have reported an effect on heart rate parameters. In humans, Steptoe^29^ reported a lower heart rate in the case of positive affect. In horses, a decrease in heart rate has also been observed in positive contexts such as grooming^30^. RMSSD (Root Mean Square of the Successive Differences) have sometimes been considered as an indicator of emotional valence (e.g. in pigs^31^ or in sheep^3^). However, the major issue with these studies is that the effect of arousal was usually not controlled, thus it is not conclusive as to whether RMSSD were a marker of valence or of arousal. When these factors were controlled, as in the study of Briefer^32^, heart rate variability was affected by arousal and not by valence. At the hormonal level, cortisol has sometimes been described as being lower following positive situations (humans^29,33^, horses^34^). Rises in oxytocin levels have also been observed in positive situations, for instance following positive interactions between a dog and its owner^33^ or following a massage session in rats^35^ and humans^36^. Nevertheless, the findings are not always clear, with opposite effects being observed. For example, a study conducted in sheep showed that oxytocin levels did not rise after a positive contact with the animal caregiver, but did increase after the animal had been left alone^37^. Further research is thus required to deepen our understanding of physiological correlates of positive affective states.

This study aimed to apply two contrasting situations in terms of emotional valence and to determine whether they were associated with specific behaviours, facial expressions and physiological responses (cortisol, oxytocin, heart rate and heart rate variability). We controlled the type of inductive stimulation by focusing only on somatosensory stimulation: tactile contacts. This type of stimulation (stroking, grooming, brushing, scratching, etc.) is interesting to study because it can generate opposite emotional responses depending on its characteristics, which can be expressed either by avoidance (cows^38,39^) or by positive responses such as contact seeking or a relaxed state (sheep^3,40^; cattle^39,41^; heifers^42^; dogs^43^; horses^15,30,44^). We thus compared two groups of horses groomed in a different manner for 11 sessions of ten minutes each. The Gentle grooming group (G, N = 13) was gently groomed and scratched focusing on the most appreciated areas, considered as being positively perceived by horses^30^. The Standard grooming group (S, N = 14) was groomed according to the standard method used in equestrian centres and taught in most horse manuals. This method is a fixed procedure that does not particularly consider the horse’s reactions. From preliminary observations, we hypothesised that these situations would lead to contrasting behavioural responses from horses towards the experimenter: contact-seeking in the first case and avoidance in the second case. We expected differences in facial expressions (position of ears, eyelids, lips and neck) according to the subjective experiences of the horses during grooming. We also hypothesised an effect of endocrine responses after the session of grooming, with a higher level of oxytocin and a lower level of cortisol in the Gentle grooming group than in the Standard grooming group. Finally, the measures of heart rate and heart rate variability should provide information regarding the level of arousal associated with each type of grooming. If grooming procedures impact specifically valence and not arousal, we would expect no change in heart rate or heart rate variability.

## Results

### Behavioural responses

All the behavioural parameters measured at S11 differed significantly between groups (Table 1). “Moving away”, “Contracting belly or back” and “Threatening/Biting” were mostly present in the S group, while “Contacts”, “Encouraging contacts” and “Attempting to nibble handler” appeared almost exclusively in the G group.

**Table 1:** Behaviours observed during S11 of grooming (number of occurrences per session).

	Gentle grooming	Standard grooming	Mann-Whitney
**Avoidance behaviours**			
Moving away	0 [0; 0]	**13 [10; 16**.**7]**	**U = 0; P < 0**.**0001**
Contracting belly or back	0 [0; 0]	**3 [2**.**25; 6]**	**U = 14**.**5; P < 0**.**0001**
Threatening or Biting	0 [0; 0]	**1 [0; 2]**	**U = 39; P = 0**.**003**
**Approach behaviours**			
Contacts	**1 [0; 5]**	0 [0; 0]	**U = 154; P < 0**.**0001**
Encouraging contacts	**8 [5; 17]**	0 [0; 0]	**U = 168; P < 0**.**0001**
Attempting to nibble handler	**4 [2; 6]**	0 [0; 0]	**U = 170**.**5; P < 0**.**0001**

Gentle grooming group, N = 13; Standard grooming group, N = 14. Mann-Whitney U test, N = 27. Median [1^st^ quartile; 3^d^ quartile]. In bold: the occurrence of this behaviour was significantly higher in this group than in the other one.

### Facial expressions

**Figure 1:**
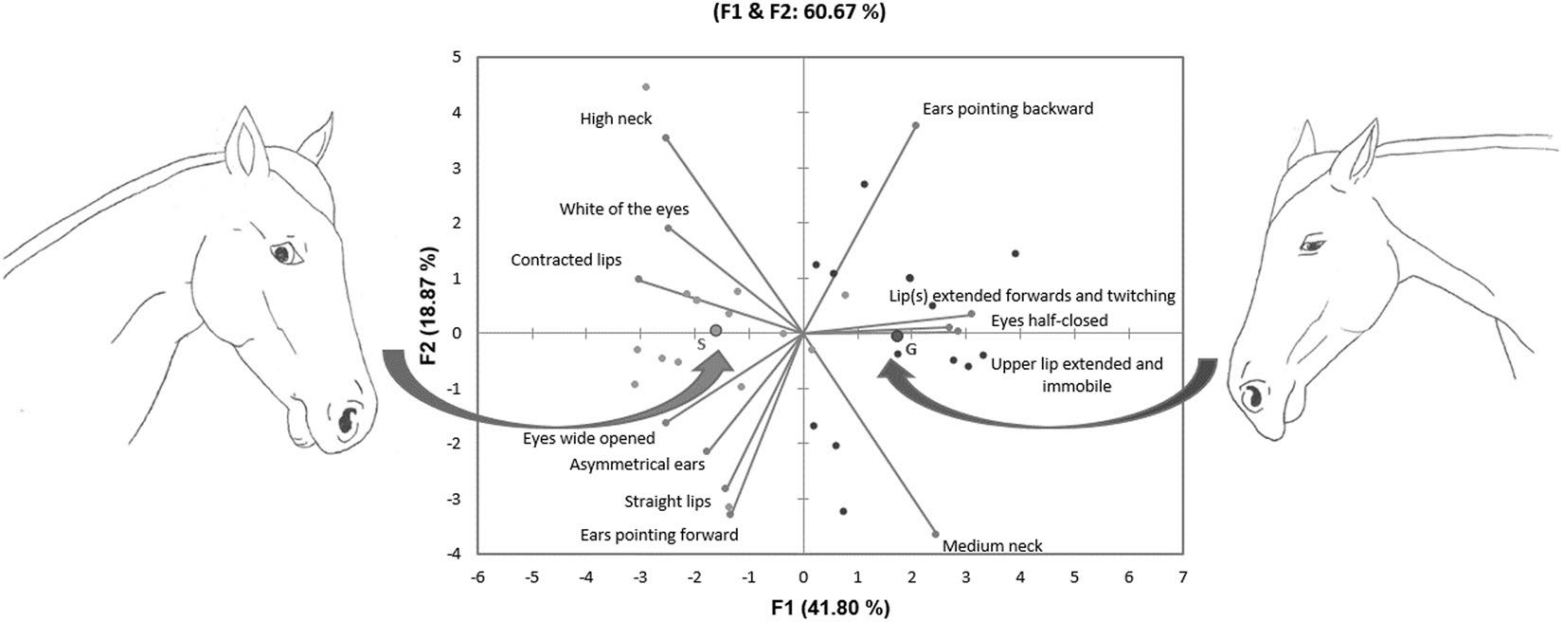
Facial expressions (PCA). This PCA was calculated with the frequency each mode occurred at S11. G: Gentle grooming group, N = 13 (dark grey points). S: Standard grooming group, N = 14 (light grey points). The two large black points correspond to the barycentre of each group.

Facial expressions were recorded during the last session of grooming (S11). Groups differed significantly for all the modes recorded (Table 2) except for “Ears pointing backwards” (Mann-Whitney U test: U = 127.5; P = 0.08), and “Ears pointing forwards” (Mann-Whitney U test: U = 86.5; P = 0.84). “Low neck” and “Ears pinned back” were not sufficiently expressed to be analysed (only 2 horses out of 27 were observed with a low neck or ears pinned back). The first two factors of the PCA, conducted to provide an overview of the facial expressions specific to each group, represented 60.67% of the total variability (Fig. 1 and Table 3). Groups were mainly represented on Factor 1 (41.80% of the total variability), with S horses mainly distributed on the left side and G horses on the right side. The scores of the subjects on Factor 1 differed significantly between groups (Mann Whitney U test: U = 177; P < 0.0001). This factor 1 opposed two groups of modes. “High neck”, “White of the eyes”, “Eyes wide opened”, “Contracted lips”, and to a lesser extent “Asymmetrical ears” were negatively correlated to the factor, and positioned on its left side. This corresponds to the typical facial expression of S horses. The modes “Medium neck”, “Eyes half-closed”, “Lip(s) extended forwards and twitching”, “Upper lip extended and immobile”, and to a lesser extent “Ears pointing backwards” were positively correlated to F1, and positioned on its right side. This corresponds to the typical expression of G horses. Factor 2 mainly opposed the different positions of ears and neck, but did not enable the two groups to be differentiated and thus is not very informative (comparison between groups of the scores on Factor 2, Mann-Whitney U test: U = 93; P = 0.94).

**Table 2:** Facial expressions observed during S11 of grooming (comparisons between groups).

Mode	Gentle grooming	Standard grooming	Mann-Whitney
High neck	0.53[0.41; 0.79]	**0**.**89[0**.**71; 0**.**94]**	**U = 38; P = 0**.**01**
Medium neck	**0**.**47[0**.**21; 0**.**59]**	0.11[0.06; 0.29]	**U = 142; P = 0**.**01**
Low neck	NA: only 2 horses/27 expressed low neck (both in G group)		
White of the eyes	0[0; 0]	**0[0; 0**.**10]**	**U = 49**.**5; P = 0**.**02**
Eyes wide opened	0.74[0.58; 0.85]	**0**.**89[0**.**78; 0**.**94]**	**U = 49**.**5; P = 0**.**04**
Eyes half-closed	**0**.**26[0**.**11; 0**.**42]**	0[0; 0.13]	**U = 143; P = 0**.**01**
Straight lips	0.55[0.42; 0.67]	**0**.**68[0**.**59; 0**.**82]**	**U = 50**.**5; P = 0**.**05**
Contracted lips	0[0; 0]	**0**.**20[0**.**14; 0**.**37]**	**U = 6**.**5; P < 0**.**0001**
Lip(s) extended forwards and twitching	**0**.**16[0**.**10 ; 0**.**33]**	0.02[0; 0.10]	**U = 150**.**5; P = 0**.**003**
Upper lip extended and immobile	**0**.**44[0**.**33; 0**.**58]**	0.05[0; 0.9]	**U = 181; P < 0**.**0001**
Asymmetrical ears	0.16[0.15; 0.26]	**0**.**33[0**.**20; 0**.**41]**	**U = 39; P = 0**.**01**
Ears pointing forwards	0.16[0.10; 0.32]	0.22[0.07; 0.28]	U = 86.5; P = 0.84
Ears pointing backwards	0.63[0.44; 0.70]	0.5[0.30; 0.55]	U = 127.5; P = 0.08
Ears pinned back	NA: only 2 horses/27 expressed ears pinned back (both in S group)		

Median [1^st^ quartile; 3^d^ quartile] of the frequency of occurrence of each mode (=number of times this mode was observed/number of observations per animal*). Mann-Whitney U test, Gentle grooming group, N = 13; Standard grooming group, N = 14. In bold: significantly higher occurrence in this group than in the other one. NA: not sufficiently expressed to be analysed.

*18.25 +− 1.05 pictures per horse and per session.

**Table 3:** Facial expressions (PCA).

	F1 (41.80%)	F2 (18.87%)
High neck	−0.676	0.634
Medium neck	0.655	−0.652
White of the eyes	−0.660	0.339
Eyes wide opened	−0.673	−0.292
Eyes half-closed	0.831	0.061
Straight lips	−0.380	−0.507
Contracted lips	−0.810	0.172
Lip(s) extended forwards and twitching	0.718	0.018
Upper lip extended and immobile	0.761	0.003
Asymmetrical ears	−0.472	−0.385
Ears pointing forwards	−0.356	−0.590
Ears pointing backwards	0.553	0.671

Correlation coefficients between the variables included in the PCA (frequency each mode occurred at S11) and the first two factors (N = 27).

### Plasma oxytocin and cortisol

**Figure 2:**
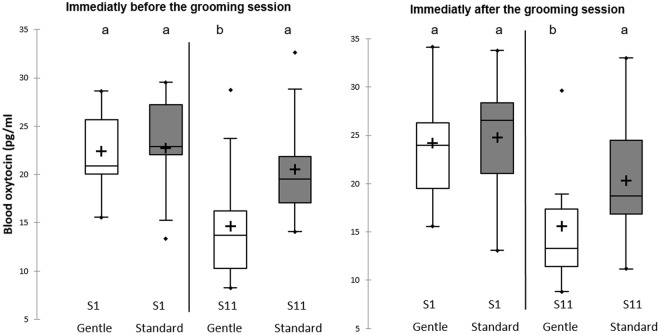
Plasma oxytocin levels. Oxytocin measured at session 1 (S1) or 11 (S11), before (on the left) or after (on the right) the grooming session. Different letters (**a**,**b**) indicate significant differences (linear mixed-effects models). Data presenting intra-assay coefficients of variation higher than 20% were excluded from the analyses (actual number of samples analysed: G, N_S1before_ = 11, N_S1after_ = 13, N_S11before_ = 12, N_S11after_ = 12; S, N_S1before_ = 13, N_S1after_ = 13, N_S11before_ = 14; N_S11after_ = 14).

The analysis of plasma oxytocin revealed non-significant differences between before and after the grooming session (linear mixed-effects models, F_1,__73_ = 1.15; P = 0.28). However, an interaction between the group and the session was found (linear mixed-effects models, F_1,76_ = 6.24; P = 0.015). Post hoc analyses revealed that oxytocin levels became significantly lower at S11 in the Gentle group while levels for the Standard group stayed constant (Fig. 2). For cortisol, no effects of the different factors tested were found (P > 0.05; Supplementary Table S1).

### Heat rate and heart rate variability

Heart rate and heart rate variability did not differ significantly between groups (Table 4).

**Table 4:** Comparisons of heart rate and heart rate variability indices between groups at session 11.

	Gentle grooming	Standard grooming	Mann-Whitney
Heart rate (bpm)	47.34 [41.40; 56.70]	51.36 [48.49; 55.82]	U = 65; P = 0.21
R-R interval (ms)	1270.41 [1069.75; 1453.30]	1175.70 [1086.37; 1249.37]	U = 117; P = 0.22
RMSSD (ms)	42.50 [34.70; 57.20]	51.35 [44.92; 57.92]	U = 77; P = 0.52
SDNN (ms)	73.50 [61.10; 92.60]	100.60 [86.20; 120.40]	U = 49; P = 0.07

median [1^st^ quartile; 3d quartile]. Mann-Whitney U test.

RMSSD: root mean square of successive inter-beat differences; SDNN: standard deviation of inter-beat intervals.

G, N = 13; S, N = 14; except for SDNN, S group N = 13.

### Long term effects of the treatment

One year later, with exactly the same grooming procedure for both groups, only one behavioural parameter was sufficiently expressed by the horses to be analysed (moving away), but it did not differ significantly between the groups (median[IQ1; IQ3] during the 2 min 30 of the test, G :0[0; 3]; S :1[0.25; 3.5]; Mann-Whitney U test: U = 40.5; P = 0.17). The other behaviours were expressed by less than 10% of the animals and were thus not analysed.

By contrast, three modalities of the facial expressions continued to differ significantly between groups: Contracted lips (median[IQ1; IQ3] of the frequency of occurrence of this mode: G:0.15[0.10; 0.33]; S:0.51[0.41; 0.62]; Mann-Whitney U test: U = 12.5; P = 0.001); Upper lip extended and immobile (G:0.03[0; 0.12]; S:0[0; 0]; Mann-Whitney U test: U = 90; P = 0.025) and White of the eyes (G:0.1[0.04; 0.16]; S:0.20[0.15; 0.20]; Mann-Whitney U test: U = 31; P = 0.05). Horses from G group had their lips extended and immobile more frequently, while S horses contracted their lips more frequently, and the white of their eyes was more visible.

## Discussion

This study aimed to apply two situations of grooming (Gentle and Standard) and to determine whether they were associated with specific behaviours, facial expressions and physiological responses. We first showed that at the end of the treatment (S11), the two types of grooming induced contrasting behaviours of approach and avoidance indicating opposite emotional valences: positive in the first case and negative in the second. We then identified very different facial expressions according to the group, both at the end of the experiment and one year later when the horses in both groups underwent an identical grooming protocol. This suggests that observing facial expressions is a very sensitive method to assess the emotions felt by an animal during grooming, even when the animals no longer express any particular behaviour. However, physiological results were not as expected: blood cortisol and oxytocin never differed between before and after the grooming session. Interestingly however, after the 11 sessions, basal oxytocin levels were lower in the G group. Finally, heart rate and heart rate variability did not differ between groups.

We used tactile stimulations to induce contrasting subjective experiences in horses. We hypothesised that an animal feeling a situation positively would seek contact, while an animal feeling a situation negatively would seek to avoid it. In line with what was expected, the horses in the “Gentle grooming” group clearly sought human contact and never expressed discomfort or defensive behaviours. In contrast, the horses from the “Standard grooming” group presented numerous discomfort, defensive and avoidance behaviours. We argue that these behaviours inform us about the animal’s emotional valence, as described in the Core affect theory^4^ (a dimensional approach differentiating a valence axis and an arousal axis). These clear behavioural differences identify two opposite emotional feelings: positive in the first and negative in the second case. These findings are very much in line with previous data which have shown that grooming can induce contact-seeking when preferred zones are massaged^30,44^, but when the horse’s reactions are not taken into account it can lead to strong avoidance and defensive reactions (preliminary observations in the field). A limit to this behavioural analysis is that the observer could not be blind to the treatment while recording the behaviours, since he could see how the experimenter was grooming the horse. Even though the behaviours recorded were easy to distinguish, and contrasted greatly between groups, we cannot exclude that this could have introduced a bias. However, this limitation did not exist for the following analyses based on the facial expressions. Indeed, these facial expressions were recorded under a blind condition, as they were based on the pictures of the head of the horses that did not allow the kind of grooming to be seen.

These blind analyses of facial expressions at S11 revealed significant differences between groups during grooming. G and S horses were clearly separated along factor 1 of the PCA. Compared to the Standard grooming horses, the Gentle grooming horses were more often observed with their necks moderately raised, their eyes half closed and their upper lips extended and either immobile or twitching and their ears pointing backwards almost in line with the nose. In contrast, horses from the Standard grooming group were more frequently observed with their neck in a high position, eyes wide open or showing the whites, contracted lips with the corner of the mouth raised jerkily and asymmetrical ears. Moreover, data show that some of these expressions are very specific to one or other of the groups (contracted lips are exclusively observed in the S group whereas eyes half closed and upper lip extended and immobile are almost only expressed in the G group), while other expressions can be observed in the two groups, but at different frequencies (e.g. high neck, eyes wide opened, straight lips or ears pointing backwards). Finally, even though we analysed the facial expressions at S11 only, it is possible that some horses from G group would have expressed the positive facial expression from the first session if they had appreciated the grooming, these expressions could in fact represent an automatic response to a positive emotion as induced by mutual grooming.

Taken together, these results suggest that facial expressions could be used to identify subjective experiences in horses in a grooming situation, and particularly positive affects. To date facial expressions in horses have mostly been described in the context of painful or stressful experiences^12,13^. However, certain features of the face have already been identified in a positive context. For example, this is the case of the angle between the line through the eyeball and the highest wrinkle which is reported to decrease during grooming in horses^19^. Our results supplement these previous observations, and show that other parts of the face, such as the position of the lips or the ears, are also of interest.

The most striking finding of this study was that one year later, despite handling both groups in a similar manner, the horses again expressed distinctive facial expressions similar to those identified in the first part of the study. These expressions were less contrasted, with fewer modalities differing significantly, but the results were very clear and in line with the observations from the previous year. The upper lip was more frequently extended forwards and immobile in the G group, and contracted with the corner raised jerkily in the S group. Regarding the eyes, they were more often wide open in group S. These results seem even more surprising considering that when analysing the behavioural elements alone nothing distinguished the two groups. The fact that the grooming procedure in year two did not take into account the horses’ reactions probably explains why the difference in behaviours between groups was less prominent than in the first year. However, the results of the facial expressions show that horses had a memory of the relationship established one year previously, despite being groomed with this intermediate procedure. Facial expressions would thus seem to be more sensitive and refined indicators of horses’ feelings than behaviours. This supports different precursory studies on animal facial expressions (e.g.^7,16,45^) and provides evidence that these can be essential in detecting emotions at least in species that have clearly distinguishable characteristic facial expressions.

This result is also important because it shows that only 11 grooming sessions induce a subjective experience in horses sufficient for them to feel different emotions a year later when handled again by the person who had previously handled them. This supports work on domestic animals, which has shown that a few handling sessions with humans can leave long-lasting traces^46^.

In future work it would be interesting to compare the facial expressions identified here with those of horses conducting mutual grooming. According to preliminary observations, they would seem to be very similar. This would indicate that horses could have the same facial expressions during interactions with both intra- and inter-specifics, such as with humans. It would be of interest in future studies to test whether horses use these expressions as a means of communication to indicate to a conspecific its intention to engage in an exchange (the case of G-type expressions), or on the contrary, to avoid doing so (type S expressions). For example, in chimpanzees it has been demonstrated that lip smacks play a role in coordinating and prolonging social grooming, a typical example of behaviour facilitating cooperation^47^. Further work could investigate possible homologies between facial expressions in different species, as has been carried out with the studies using FACS (e.g.^24,45^). For instance, it is fascinating to notice certain similarities between the lip smacking of chimpanzees and extended lips of horses. Moreover, the ear position directed backwards in the G group is interestingly also found in sheep when being stroked^40^. The fact that several species can show similar expressions, could be explained by their similar evolutionary pattern. For instance, lip movement could stem from mutual grooming itself, which involves mouth movements and which could have become ritualised with this more discrete lip movement. In any case, it would seem reasonable that these facial expressions would have a communicative use at least between conspecifics, but even possibly between species.

Regarding the endocrine responses, contrary to what was expected, we did not find any changes after either type of grooming. No rise in cortisol was observed in horses groomed in a rough manner despite their avoidance behaviours and their specific facial expression. It is possible that the situation of standard grooming was not stressful enough for the horses to induce a rise in cortisol, unlike what can be observed in horses after social isolation, a novel environment or stressful husbandry procedures^28,48,49^. Cortisol level did not decrease after the gentle grooming either, as could have been expected if it was a marker of positive emotions as suggested in some studies (humans^29,33^, horses^34^). Our finding does not support the hypothesis that tactile stimulation via gentle grooming would elicit an increase in peripheral oxytocin levels. We posited this hypothesis because many studies have reported an oxytocin release in response to tactile stimulation in adult rats^50^ and humans^36^ and also during positive social contact including touch when dogs interact with their owner^51^ or in humans when parents and infants interact^52^. The lack of consistency with our results may reflect differences in experimental situations or sampling procedures. In particular, the relevance of measuring oxytocin at the plasma level has sometimes been questioned, as brain oxytocin neuropeptide is considered to be a much more reliable candidate^53^. In lambs which have been able to develop a strong bond with a human, oxytocin plasma levels are found to decrease, rather than increase, when they reunite and interact with their human caregiver after a period of social isolation^37^. By contrast, within the brain, oxytocinergic neurons are activated in the paraventricular nucleus of the hypothalamus in lambs that are able to interact with their caregiver compared to lambs which remain isolated^54^. Indeed, when studying oxytocin it is important to choose carefully the method used to investigate it^55^. It does not mean that peripheral oxytocin is irrelevant and our study shows that plasma levels were affected by our treatment. Indeed, we found an unexpected but interesting result: oxytocin levels became significantly lower at S11 in the Gentle group, both before and after the session. An explanation would be that the basal levels of oxytocin might have been lowered in the G group horses because the repetition of gentle grooming sessions may have induced a general state of well-being in the animals, which was not the case at the start of the handling sessions. On the other hand, horses groomed in a standard manner would not have reached this well-being state because the treatment had maintained a more negative emotion. Such a physiological state is observed in prairie voles that are subjected to chronic social isolation^56^ or in lambs reared without a mother^57^. In humans, higher basal levels of oxytocin in plasma have also been associated with greater relational distress^36,58–60^. In addition, it is known that oxytocin has an anxiolytic and anti-stress effect, and can be more elevated in periods of distress, thus down regulating the hypothalamic-pituitary-adrenal axis^58,61^. This could also be the case in horses, but further research is required to confirm this result and understand the underlying mechanisms.

Finally, regarding the heart rate and heart rate variability, considered as markers of emotional arousal^32,62,63^, there were no statistical differences between groups. In contrast with studies in horses showing that positive stroking could have a calming effect and thus lower heart rate parameters^30^, there is no conclusive evidence of a relaxing effect due to stroking in the G group. This is in line with the behavioural observations that suggest a relatively high level of arousal in both groups. Indeed, horses presented active behaviours in both groups: S horses showed avoidance behaviour while G horses showed seeking behaviours. Horses also had tense facial expressions in both groups. In fact, G horses did not present relaxed faces like those observed during rest with the lower lip hanging in a relaxed way, but instead they had tense faces with ears pointing backwards and the upper lip extended forwards. Thus, taken together, these results suggest that on the Core affect graph^4,64,65^, the two groups could be distinguished on the emotional valence axis while being relatively similar on the arousal axis.

To conclude, this study demonstrated that horses presented contrasting behaviours according to the kind of grooming experienced (Gentle vs Standard), suggesting opposite emotional valences, and that specific facial expressions were associated with them. These expressions appear to be more sensitive than behavioural indicators because they alone enabled different emotions to be detected according to the group one year after the end of treatment. In line with previous studies, this new work confirms that research on facial expressions is a promising avenue in the field of animal emotions. In the present study, the facial expressions identified were probably specific to the context of grooming, and a lot of other facial expressions remain to be identified in different contexts and for different emotional valences and/or levels of arousal. Regarding endocrine responses, despite the unexpected results, the differences in basal oxytocin levels observed between groups at the end of treatment lead the way to further research to confirm whether or not basal oxytocin could also be a marker of well-being in horses. Finally, this study has shown to what extent grooming practices, which could appear to be inoffensive, have a long-term incidence on the horse’s general well-being and the human/horse relationship. This study thus serves as a basis to promote respectful and safe animal welfare practices in the field based on careful observations of behaviours and facial expressions.

## Methods

### Ethics statement

The protocol was approved by the ethics committee of Val de Loire (APAFIS#40 13–20 1602091626908 delivered by CEEA Val de Loire). Animal care and experimental treatments complied with the guidelines of the French and European guidelines for housing and care of animals used for scientific purposes (European Union Directive 2010/63/EU) and were performed under authorization and supervision of official veterinary services (agreement number F371752 delivered to the UEPAO animal facility by the veterinary service of the Département d’Indre et Loire, France).

### Animals and housing conditions

The study was conducted on 27 Welsh mares, aged 2 (n = 19) or 3 years old (n = 8). From birth until the beginning of the experiment they lived in large groups at pasture during summer and in large inside stalls all together with access to a paddock in winter. They were fed ad libitum with straw and hay. They were checked daily for their general state, but never groomed.

### Groups and general conditions of grooming

From three months before the beginning of the experiment to one week before, all horses were handled twice a week for five minutes to habituate them to a halter, and to being attached in a loose box and touched all over their bodies. Nine days before the beginning of the experiment, horses were tethered for five minutes a day in the testing arena (see below). This initial handling phase was carried out by a person who was not involved in the experiment. The horses were then randomly divided into two groups, balanced for age and weight: Gentle grooming (G, N = 13) and Standard grooming (S, N = 14). For the two weeks of treatment, the two groups lived together at pasture at night. From 9:00 am until 4:00 pm, they were housed in triads in large loose boxes (5.5 × 3.5 m) combining horses from each group. After the experiment, they returned to pasture with exactly the same husbandry routine as before.

Horses were handled by the same female experimenter for eleven 10-min sessions of grooming per day (S1 to S11). These sessions were conducted on six consecutive days followed by one day off, and then on five more consecutive days (Supplementary Figure S1). For each grooming session, horses were fitted with a halter in their box, led to the testing arena (5.5 × 3.5) located in the same barn and tethered with two leading reins in the middle of the arena. To avoid any stress due to social isolation, they were in visual contact with a familiar horse, tethered in a loose box 5 m in front of them. This horse was always the same, and was not part of the experimental treatments. The other horses could not see inside the testing arena and thus could not observe the grooming procedure. The arena was equipped with two video cameras: one recorded the whole body of the horse (Sony, DCR-SR21E), and the other (Sony, HRD-PJ410) focused on the head. Two observers were positioned behind a wall equipped with a metal grating enabling them to watch the horse without being seen. One of them recorded the behaviours while the other managed the video cameras.

### Standard grooming

The experimenter groomed each horse in this group for 10 minutes per session with a standard procedure, that had been established during a previous series of observations made in the field on 69 different horse/rider couples, and which had been shown to induce numerous avoidance reactions. During these previous observations, for each horse we had recorded the kind of brush used, the pressure put on the brush, the frequency of strokes per minute and the duration each brush was used. Means were calculated for the duration and the frequency of the use of each brush. The present protocol was based on these observations. The experimenter began systematically on the left side of the horse, and groomed in the following order, the neck, head, shoulder, back, flank, belly, hindquarters and finally the legs. The procedure lasted 10 min and involved grooming each side of the horse first with a currycomb for 1 min 40 (except for the head and the legs), then with a hard-bristled dandy brush for 2 min 12 and finally with a soft body brush for 1 min 08. Each brush stroke was of medium intensity covering around 30 cm with approximately 80 strokes per minute. This standardized procedure was maintained independently of the horse’s reaction.

### Gentle grooming

The experimenter groomed each horse in this group for 10 minutes per session with a procedure which aimed to maximise positive reactions and minimise negative reactions. During each session, grooming began on the left side of the horse and lasted for 4 minutes, and involved using the hands to scratch the horse’s neck, then head, back, belly and hindquarters, and finishing on each leg. When the horse demonstrated approach behaviours (Table 1) regarding a particular area, the experimenter focused on this zone for longer and increased the pressure. When the horse showed no reaction for 30 s or showed an avoidance reaction, the experimenter decreased the pressure and changed to another zone. After this she brushed each part of the body for a minute with a plastic currycomb with small circular movements, decreasing the pressure at the slightest sign of avoidance behaviour. This procedure was repeated on the other side of the horse, beginning this time by brushing with the currycomb for a minute, and finishing with the 4 minutes of scratching with the hands. Thus, at the end of each session, horses from each group had been groomed for the same duration, on exactly the same parts of their body, but in a different way.

### Long term effects of the treatment

At the end of the treatment, horses returned to their normal housing (see section ‘Animals and housing conditions’), and were no longer groomed. A year later, 10 S horses and 12 G horses were tested again by the same experimenter during a session of 2 min 30.

Since the aim was to determine whether the horses had a memory of the grooming sessions conducted one year previously, we choose to carry out a short session (2 min 30 instead of 10 min), to minimise the influence of a new training effect. During this session, horses were tethered with two leading reins in the middle of the testing arena (equipped in the same manner as described in the section ‘general conditions of grooming’). The grooming procedure was the same for both groups and was intermediate to those used previously in the two treatments. The experimenter gently brushed the different zones of the horse’s body with a plastic currycomb, in the same order as detailed above. No pressure was applied with the brush. The procedure was not adapted according to the horse’s positive or negative reactions, and did not involve scratching preferential zones.

### Parameters recorded: behavioural responses

The horses’ behaviours were recorded continuously (all occurrences method), directly by the same observer during the 11^th^ session of grooming and during the single test carried out a year later (Supplementary Figure S1). The behavioural repertoire consisted of two behavioural categories: avoidance behaviours and approach behaviours (Table 5). In the case of a behaviour lasting more than three seconds without interruption, the observer noted its occurrence every three seconds.

**Table 5:** Behavioural repertoire recorded during grooming sessions.

Avoidance behaviours	
Moving away	Horse moves in the opposite direction to the handler’s action
Contracting belly or back	Horse contracts its belly or back suddenly after a brush stroke
Threatening or biting	Horse’s ears are pinned back and a hind leg is lifted in the direction of the handler or Horse’s ears are pinned back and lips are pulled back showing the teeth in the handler’s direction, the horse tries to bite the air or the leading rein
**Approach behaviours**	
Contacts	Horse seeks contact with the handler with its head, without signs of threatening or biting
Encouraging contact	Horse moves part of its body to lean or rub against the handler, sometimes with a backward and forward movement
Attempting to nibble handler	Horse’s upper lip is extended and mobile, horse nibbles the handler or any other element in front of it (wall, leading rein, etc.)

### Parameters recorded: facial expressions and height of neck

We analysed the positions of the neck, eyelids, lips and ears from the video footages from session 11 and from the single test carried out a year later (Supplementary Figure S1 and Table 6). From each video, we took 20 freeze frames per animal. As the length of the session varied between the period of treatment in year one (length: 10 min) and the test performed in year two (length: 2.5 min), one freeze frame was taken every 30 seconds in year one or every 7 seconds in year two. When the head had moved at this moment and was out of focus or the face was not visible, the frame was delayed for a few seconds. In this case, the following frames were taken at the scheduled times. We then removed images of poor quality where all the parameters could not be observed correctly. At the end, the total number of images analysed per horse was 18.25 + 1.05 per session (range: 15 to 20). For each image, a single observer (OBS 1) attributed a mode corresponding to each part of the head, according to the descriptions in Table 6. The observer was blind to the treatment. This was possible as the images did not enable the observer to see how the experimenter groomed the horse. This observer was not involved in the handling procedure or in recording the behaviours. The modes that were recorded were chosen in accordance with the literature which existed prior to the experiment^12^, and from three pilot studies which aimed to describe for the different parts of the horse’s head the different modes that are expressed in different contexts (grooming, social separations, rest, riding activity, etc.). In each case, at least two observers recorded the modes from video footage. One was naïve (trained in ethology, but who had never observed horses before), while the other was accustomed to observing horses. Only the measures that present good reliability between the observers were selected and reused in the present experiment. Moreover, to check the inter-observer reliability, 20% of the data of the present experiment were reanalysed by a second observer (OBS 2), also blind to the treatment (a sample of 100 images: 4 images per horses randomly chosen during the session, on 25 horses). The level of observers’ agreement on the facial expressions was high (Cohen’s Kappa and percentage agreement for Height of neck: 1 and 100%; Opening of eye: 0.97 and 99%; Lip tension: 0.88 and 93%; Position of ears: 0.92 and 95%).

**Table 6:** Facial expressions and height of neck recorded during grooming sessions. Modes associated with each part of the head.

Part of the head	Modes	Description
Height of neck^a^	High Medium Low	Angle between neck and withers of 130 to 165° Angle between neck and withers of 166 to 201° Angle between neck and withers of 202 to 237°
Opening of eye	White of the eyes Eyes wide opened Eyes half closed	Sclera visible and eyes wide open Eyelids completely open Eyelids only slightly open
Lip tension^b^	Straight lips Contracted lips Lip(s) extended forwards and twitching Upper lip extended and immobile	No tension in the lips and they are aligned with each other Lips are contracted. Corner of upper lip is contracted and raised jerkily Upper or/and lower lip are extended and twitch or move laterally or vertically Upper lip is extended and immobile, lower lip is immobile
Position of ears	Asymmetrical ears Pointing forwards Pointing backwards Pinned back	Ears oriented in different directions (forwards, backwards or to the side) Ears oriented forwards Ears oriented backwards, almost aligned with the nose, in a fixed position Ears pinned totally back in the mane

^a^The angle between the neck and withers was measured on the image using a protractor (130° was the lowest angle observed while 237° was the highest).

^b^See Supplementary Figure S2 for pictures of the lips.

### Parameters recorded: blood oxytocin and cortisol

Blood samples were taken before the horse was led into the testing arena, and then immediately at the end of the grooming session in the testing arena for the first and last grooming sessions (S1 and S11 respectively, Supplementary Figure S1). Blood samples (20 ml) were taken from the jugular vein by two experienced people who were not involved in any other part of the experiment. The procedure was carried out calmly, lasted less than a minute and did not cause any reaction in the horse. The blood was stored at −20 °C until analysis. Oxytocin and cortisol assays were performed and replicated in the INRA laboratory, Nouzilly, France. The experimenter was totally blind to the treatment. For oxytocin an ELISA (kit ADI-901–153, Enzo Life Sciences) was performed after molecular extraction using Sep-Pak C18 cartridges (Waters). Cortisol was assayed using radio immunology (RIA) after steroid extraction with a solvent (ethyl acetate/cyclohexane). Replicate measurements were performed. The intra-assay coefficients of variation were 17% at 20 ng/ml for cortisol, and 1.5% at 100 pg/ml for oxytocin. The assay sensitivities were 0.5 ng/ml and 7.8 pg/ml for cortisol and oxytocin respectively. Data presenting intra-assay coefficients of variation higher than 20% were excluded from the analyses.

### Parameters recorded: Heart rate and heart rate variability

The mean heart rate and heart rate variability (RR interval, RMSSD, SDNN) were recorded at S11 during the 10-min grooming session (Supplementary Figure S1). The heart monitor system (Polar Equine RS800CX Science, Polar Oy, Finland) consisted of a flexible belt with two integrated electrodes, a transmitter, a separate storage device, and the corresponding software (Polar Pro Trainer, Version 5). The electrodes were placed behind the left humerus and across the sternum, and electrical conductivity was enhanced using ultrasound gel. The storage device was fixed to the belt within range of the transmitter. Animals were habituated to the device three times during the week preceding the beginning of the experiment.

### Statistical Analysis

For each horse, the number of times each behaviour was expressed during the 11^th^ grooming session and during the single test carried out a year later was calculated. The two sessions were analysed separately. For the facial expressions, we calculated the frequency at which each mode occurred in all the freeze frames, for each horse and each year (S11 and test carried out a year later). For instance, when the horse’s ears were observed N times in the “asymmetrical ears” position in the 20 images taken during S11, the frequency for the mode “asymmetrical ears” for that session was N/20. Due to the number of animals and the distribution of the data, non-parametric statistics were used to analyse the behavioural data, facial expressions, heart rate and heart rate variability (Mann-Whitney U test for inter-group comparisons). Two-tailed tests were always used. Data are presented as median [1^st^ quartile; 3^d^ quartile]. The facial expressions (frequency of occurrence of each mode) were also analysed using a Principal Component Analysis (PCA) to give an overview of potential facial expressions specific to each group. In this PCA, the different modes of facial expressions were analysed as active variables and the groups as illustrative variables. Two modes (“Low neck” and “Ears pinned back”) were not included in the PCA because they were expressed by hardly any animals (only 2 horses out of 27 were observed with a low neck or ears pinned back). Due to the distribution of the data, the PCA was based on Spearman correlations. The scores of the subjects of each group on the first two factors of the PCA were also compared with a Mann-Whitney U test. These analyses were performed using Xlstat software (Addinsoft, Paris, France) with significance accepted at P ≤ 0.05. We present the original P values because in cases of low sample size, the usefulness of using corrections for multiple comparisons is highly debated and results in a loss of power^66–68^.

Oxytocin and cortisol were analysed with linear mixed-effects models (lmm) in R (version 3.3.2, R Development Core Team, 2016) using the lmer function from the lme4 package. Models were fitted with restricted maximum likelihood estimation (REML) and p-values were derived using the Satterthwaite approximations (anova function from the lmerTest package). These models included oxytocin or cortisol as a response variable, the session number (S1 or S11), the factor "before or after session" and the treatment group (G or S) as fixed factors, as well as all possible interactions between them. The individual identity of the horses was included as a random factor. Non-significant interactions were removed from the final models (for oxytocin, this was the case of the interactions between groups x before/after, and session number x before/after; for cortisol, no factors or interactions were significant). Residuals were checked graphically for normal distribution and homoscedasticity. Post hoc analyses were conducted using a Tukey’s test.

The datasets generated and analysed during the current study are available from Zenodo (10.5281/zenodo.1321000).

## Electronic supplementary material

Supplementary information
