# Supplementary material for: Facial expression and oxytocin as possible markers of positive emotions in horses
Source: Sci Rep. 2018 Oct 2;8:14680. doi: 10.1038/s41598-018-32993-z (PMC6168541; doi:10.1038/s41598-018-32993-z)
Supplement: Supplementary file 1 — Supplementary information [file 41598_2018_32993_MOESM1_ESM.pdf]

Facial expression and oxytocin as possible markers of positive emotions in horses

Lansade Léa [a]*, Nowak Raymond [a], Lainé Anne-Lyse [a], Leterrier Christine [a], Bonneau Coralie [a], Céline Parias [a], Bertin Aline [a]
Affiliations: [a] PRC, INRA, CNRS, IFCE, University Tours, 37380 Nouzilly, France

*Corresponding author

mail: lea.lansade@inra.fr

phone: 00 33 2 47 42 72 79

Supplementary information

	Gentle grooming	Standard grooming
S1, before handling	64.61[61.41;81.31], N=13	70.43[44.21;82.62], N=14
S1, after handling	67.16[60.98;74.06], N=11	70.42[55.38;87.72], N=11
S11, before handling	64.05[51.87;70.73], N=13	59.10[46.75;85.15], N=14
S11, after handling	74.55[67.81;78.56], N=12	72.52[50.04;92.25], N=14

Supplementary Table S1. Blood cortisol levels (ng/ml) observed before and after the handling session Median [1st quartile; 3d quartile]. Data presenting intra-assay coefficients of variation higher than 20% were excluded from the analyses. The actual number of samples analysed are presented in the table. Groups, sessions and the time of sampling (before or after the handling session) had no significant effect (linear mixed-effects models, P>0.05).

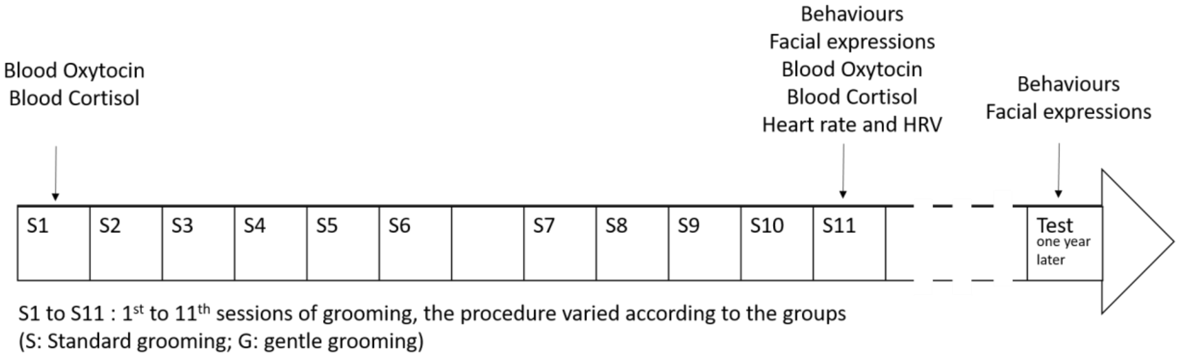

Supplementary Figure S1. Timeline of the experiment

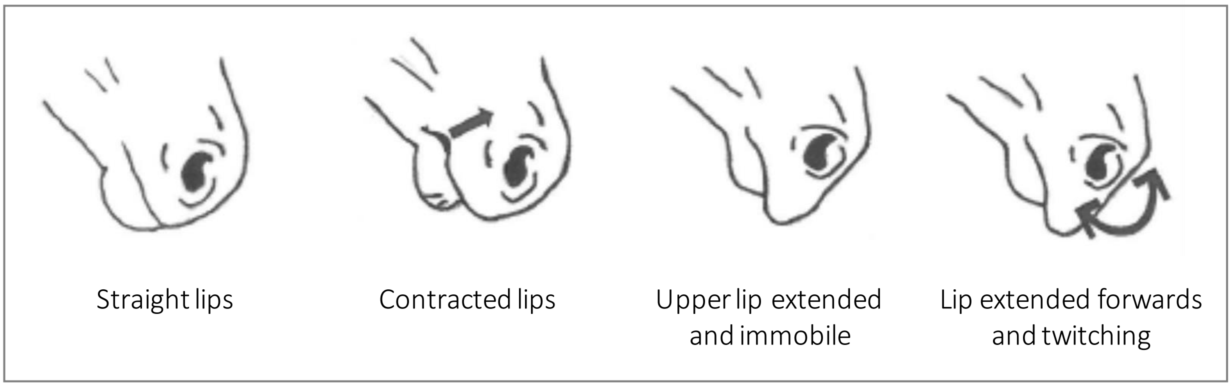

Supplementary Figure S2. Lip tension
